# Patients with cocaine use disorder exhibit reductions in delay discounting with episodic future thinking cues regardless of incarceration history

**DOI:** 10.1016/j.abrep.2023.100518

**Published:** 2023-10-30

**Authors:** Taylor M. Torres, Stuart R. Steinhauer, Steven D. Forman, Sarah E. Forster

**Affiliations:** a VA Pittsburgh Healthcare System, VISN 4 Mental Illness Research, Education, & Clinical Center (MIRECC), USA; b University of Pittsburgh, Department of Psychiatry, USA

**Keywords:** Addiction, Cocaine use disorder, Delay discounting, Episodic future thinking, Incarceration

## Abstract

Research examining episodic future thinking (EFT; i.e., imagining oneself in future contexts) in community samples has demonstrated reduced discounting of delayed rewards when personalized event cues are included to prompt EFT related to reward latencies. While this EFT effect was recently demonstrated in individuals with substance use disorders, it is not yet known if it manifests similarly in individuals with and without a significant incarceration history—the latter being at elevated risk for negative outcomes including criminal recidivism. Individuals with cocaine use disorder (n = 35) identified personally-relevant future events and participated in a computerized delay discounting task, involving decisions between smaller immediate rewards or larger delayed rewards with and without EFT cues. Individuals with (n = 19) and without (n = 16) a significant history of incarceration were identified using the Addiction Severity Index-Lite. A significant reduction in discounting rates was observed when event cues were included to promote EFT (p = 0.02); however, there was no main effect of incarceration history on discounting behavior, or interaction between episodic future thinking condition and incarceration history. Results suggest personalized cues included to evoke EFT reduce discounting behavior in individuals with cocaine use disorder, regardless of incarceration history. EFT–based interventions may therefore have promise to reduce impulsive decision-making in individuals with cocaine use disorder with and without a significant history of incarceration, potentially supporting improved outcomes with respect to both substance use and future criminality.

## Highlights

• Episodic future thinking (EFT) has been shown to reduce impulsive decision-making.
• This EFT effect has previously been demonstrated in substance use disorders (SUDs).
• No previous work has examined EFT with respect to incarceration history.
• Findings show no impact of incarceration history on the EFT effect in SUD patients.
• EFT-based interventions may be useful in SUDs, regardless of incarceration history.

## 1 Introduction

Choosing immediate gratification over delayed reward is a common decision. However, the rate at which future reward loses value depends upon individual differences (Odum, 2011). Psychiatric conditions characterized by behavioral disinhibition (e.g., Substance Use Disorders (SUDs), Attention-Deficit/Hyperactivity Disorder, Borderline Personality Disorder) are associated with a tendency to favor immediate gratification (Levitt et al., 2020). Delay discounting (DD), the phenomenon whereby a reward’s subjective value decreases with delays, measures this tendency—with more impulsive individuals exhibiting steeper discounting rates, (i.e., reward loses value more rapidly with increasing temporal distance). Importantly, DD is associated with real-world behaviors in community samples, as well as patient populations characterized by pathological disinhibition. For example, steeper discounting rates have been linked to greater reactive thinking (i.e., emotional-impulsive thought processes) in formerly incarcerated adults (Varghese et al., 2014), which appears to mediate the relationship between past criminal behavior and future offending (Walters, 2016).

As an analogue for real-world impulsive behavior, DD provides a proving ground for novel intervention strategies, of which episodic future thinking (EFT) manipulations appear especially promising (Rung and Madden, 2018, Scholten et al., 2019). EFT, imagining oneself in future contexts, may reduce impulsive decision-making by increasing the psychological proximity of delayed rewards. These mental simulations combine sensory details drawn from episodic memories with personal semantic information (Schacter et al., 2017), with the latter playing a more integral role with increasing temporal distance (La Corte & Piolino, 2016) and supporting variably idealized projections of the future self (Sellitto, 2020). In DD, EFT has been evoked by including cues or prompts referencing personally–relevant future events (e.g., plans, goals, anniversaries) and has been shown to reduce discounting rates in community samples and clinical populations, including SUDs (Bickel et al., 2016, Rung and Madden, 2018, Scholten et al., 2019). Importantly, such EFT manipulations have been shown to support future-conscious, real-world behaviors, such as reduced tobacco use and food intake (see Scholten et al., 2019), and may similarly have implications for substance use relapse and criminal recidivism.

While reduced discounting under EFT conditions has been demonstrated in SUD populations, including alcohol, cannabis, and cocaine use disorder (CUD; Forster et al., 2021, Mellis et al., 2019, Snider et al., 2016, Sofis et al., 2020), it has not yet been evaluated relative to personal incarceration history. Similar to individuals with SUDs, previously incarcerated individuals exhibit higher trait impulsivity (Pratt & Cullen, 2000), which contributes to diminished future orientation and steeper DD. Indeed, incarcerated individuals display more pronounced DD than non-offenders, even when controlling for substance use history (Arantes et al., 2013). Research on recently released inmates has further shown steeper DD among individuals with higher rates of reincarceration (Varghese et al., 2014). As substance use history has been associated with even greater impulsivity among incarcerated individuals (Cuomo et al., 2008), the effectiveness of EFT manipulations in individuals with a history of both SUD and incarceration is of special interest.

To examine whether the EFT effect on DD varies by incarceration history in individuals with SUDs, we utilized a personalized DD paradigm to measure discounting behavior with and without cues to evoke EFT. Participants were treatment-seeking individuals with CUD, with approximately half the sample reporting a history of incarceration. It was hypothesized that EFT cues would reduce discounting behavior in individuals with CUD, regardless of incarceration history.

## 2 Methods

### 2.1 Participants

Participants were treatment-seeking U.S. military Veterans, aged 18–75, diagnosed with CUD and recruited through the VA Pittsburgh Healthcare System (VAPHS). All participants provided written, informed consent to participate, as approved by the VAPHS Institutional Review Board, and reported cocaine use within the past 60 days, as well as normal or corrected-to-normal a.udiovisual acuity. Exclusion criteria included history of severe traumatic brain injury, neurological disease, or convulsive therapy, and moderate-to-severe cognitive impairment. Participants tested negative for non-prescribed, controlled substances via urine- and/or saliva-based point-of-care testing and negative for alcohol via breath- or saliva-based testing prior to assessment. Compensation was provided for time and effort. Thirty-five individuals with CUD (91% male) were included, with 19 participants reporting a history of incarceration and 16 participants reporting no incarceration history. Additional demographic and clinical characteristics are presented in Table 1.

**Table 1:** Discounting Task Data and Sample Characteristics (M (SD) or N (%)).

Variable	Category or Group	Incarceration History (n = 19)	No Incarceration History (n = 16)
*Age*		62.6 (5.6)	58.5 (7.8)
*Past Month Stimulant Use*		111.1 (117.7)	0 (0)
*Past Month Alcohol Use*		7.1 (7.6)	4.5 (4.7)
*Past Month Other Drug Use*		3.8 (4.1)	4.3 (4.2)
log*(k) EFT*		−3.22 (2.51)	−3.12 (2.67)
log*(k) Standard*		−2.27 (3.33)	−2.41 (3.11)
*Event Latencies EFT*		2.5 (5.9)	3.9 (7.5)
*Event Latencies Standard*		6.7 (11.7)	4.3 (8.9)
*Event Rating: Relevance*		6.4 (0.97)	6.3 (1.1)
*Event Rating: Positivity*		6.4 (0.99)	6.3 (1.0)
*Event Rating: Excitement*		6.3 (1.1)	6.1(1.2)
*EFT Vividness Rating*		4.6 (1.4)	4.9 (1.3)
*Gender*	Male	18/19 (94.7%)	14/16 (87.5%)
	Female	1/19 (5.3%)	2/16 (12.5%)
*Race*	White or Caucasian	2/19 (10.5%)	3/16 (18.7%)
	Black or African American	17/19 (89.5%)	13/16 (81.3%)
*Ethnicity*	Hispanic or Latino	2/19 (10.5%)	0/16 (0%)
	Not Hispanic or Latino	17/19 (89.5%)	16/16 (100%)
*Mental Health Diagnoses*	Post-Traumatic Stress Disorder	5/19 (26.3%)	4/16 (25%)
	Generalized Anxiety Disorder	2/19 (10.5%)	3/16 (18.7%)
	Bipolar and Related Disorders	6/19 (31.6%)	3/16 (18.7%)
	Major Depressive Disorder	8/19 (42.1%)	6/16 (37.5%)
	Antisocial Personality Disorder	3/19 (15.7%)	4/16 (25%)
	Panic Disorder	1/19 (5.2%)	1/16 (6.25%)
	Agoraphobia	1/19 (5.2%)	1/16 (6.25%)
	Psychotic Disorders	3/19 (15.8%)	3/16 (18.8%)
	Social Phobia	0/19 (0%)	3/16 (18.7%)
	None	1/19 (5.3%)	1/16 (6.25%)
*Secondary SUD Diagnoses*	Alcohol Use Disorder	9/19 (47.3%)	7/16 (43.7%)
	Opioid Use Disorder	4/19 (21%)	3/16 (18.7%)
	Cannabis Use Disorder	4/19 (21%)	4/16 (25%)
	Inhalant Use Disorder	0/19 (0%)	1/16 (6.25%)
	None	7/19 (36.8%)	4/16 (25%)
*Housing Status*	Independent Housing	14/19 (73.6%)	13/16 (81.4%)
	Personal Care Home (PCH)	1/19 (5.3%)	0/16 (0%)
	Transitional Housing	1/19 (5.3%)	1/16 (6.2%)
	Residential Treatment Setting	1/19 (5.3%)	1/16 (6.2%)
	Unstable / Homeless	2/19 (10.5%)	1/16 (6.2%)
*Employment Status*	Employed full-time	1/19 (5.3%)	4/16 (25%)
	Employed part-time	1/19 (5.3%)	0/16 (0%)
	Unemployed	6/19 (31.5%)	2/16 (12.5%)
	Retired or Disabled	11/19 (57.9%)	7/16 (62.5%)
*Event Latencies:* *EFT / Standard*	1 Week	4.8 (1.8) / 5.9 (2.6)	5.3 (2.1) / 5.8 (2.3)
	2 Weeks	13.9 (2.9) / 13.9 (3.6)	15.1 (2.8) / 14.6 (3.4)
	1 Month	33.1 (7.3) / 33.7 (8.9)	36.0 (6.4) / 36.8 (9.1)
	3 Months	94.6 (17.5) / 91.0 (15.8)	91.9 (14.4) / 90.4 (13.1)
	6 Months	192.6 (22.2) / 195.7 (29.7)	194.6 (26.2) / 195.8 (27.9)
	1 Year	352.1 (33.3) / 344.1 (43.5)	345.6 (26.1) / 344.5 (33.3)

### 2.2 Procedures and measures

Participants completed assessments prior to randomization into a trial of Contingency Management at VAPHS (see Forster, Forman, et al., 2021, for details). Assessments included the Addiction Severity Index-Lite, wherein incarceration history was queried. Methods for the personalized DD task were generally as described in Forster, Steinhauer, et al. (2021). In brief, an interview was conducted to identify positively- or neutrally-valenced, personally-meaningful future events at latencies approximating 1-week, 2-weeks, 1-month, 3-months, 6-months, and 1-year from the testing date. As previously described (see Forster, Steinhauer, et al., 2021) and in contrast to other similar work (Chiou and Wu, 2016, Mellis et al., 2019, Snider et al., 2016, Sofis et al., 2020) episodic specificity induction techniques were not utilized to support EFT during the interview or computerized cognitive task.

Events were rated on a 7-point Likert scale for personal relevance, valence, and arousal/excitement (see Supplementary Materials) and included in a personalized DD task, programmed using E-Prime (Psychology Software Tools, Pittsburgh, PA) and administered via desktop computer. On each trial, participants decided between $10 available “today” or a different amount, available at some delay. Details of immediate and delayed options were summarized on the left and right side of the display, respectively (see Supplementary Fig. 1), and participants responded by pressing the corresponding left- versus right-most button on a response pad.

Twelve blocks of trials, representing two conditions (EFT, Standard) and 6 target latencies, were presented in a randomly-determined order. Latencies for the EFT condition reflected dates of future events identified. Improving upon our previous work (Forster, Steinhauer, et al., 2021), Standard latencies were pseudo-randomly determined to approximate EFT latencies and ensure conditions were matched for chronological primacy. A staircase algorithm determined delayed values to systematically approximate the “switch point” at which preference shifted to the larger, delayed reward (maximum amount: $50). A more rigorous control for the EFT effect was also implemented by displaying dates for Standard latencies in place of personally-relevant event descriptors used in the EFT condition (see Discussion). Additional minor methodological differences, not directly relevant to current findings, will be detailed elsewhere.

### 2.3 Data analysis

Determination of “switch points,” modeling of DD functions, and evaluation of model fit were conducted using methods previously described in Forster, Steinhauer, et al. (2021). We additionally implemented a modified version of the algorithm for detection of non-systematic DD responses described by Johnson & Bickel (2008; see Supplementary Materials). Because EFT cues introduce variability in response contexts across latencies (potentially engendering non-systematicity), results are presented with and without omission of these datapoints. The discounting rate parameter (*k*) was computed separately for EFT and Standard conditions for each participant. A natural log transformation was applied due to skewness in the distribution of *k* values, yielding log(*k*). Due to the non-normal distribution of the data, a non-parametric Aligned Rank Transform (ART) repeated-measures analysis of variance (rANOVA) was used for within (EFT, Standard) and between (incarceration history, no incarceration history) group statistical tests (Wobbrock et al., 2011) and *post hoc* comparisons (Elkin et al., 2021). Descriptive statistics further summarize subjective ratings, individually-determined task and discounting model parameters, and relevant participant characteristics for incarcerated and non-incarcerated subgroups (see Table 1).

## 3 Results

**Fig. 1:**
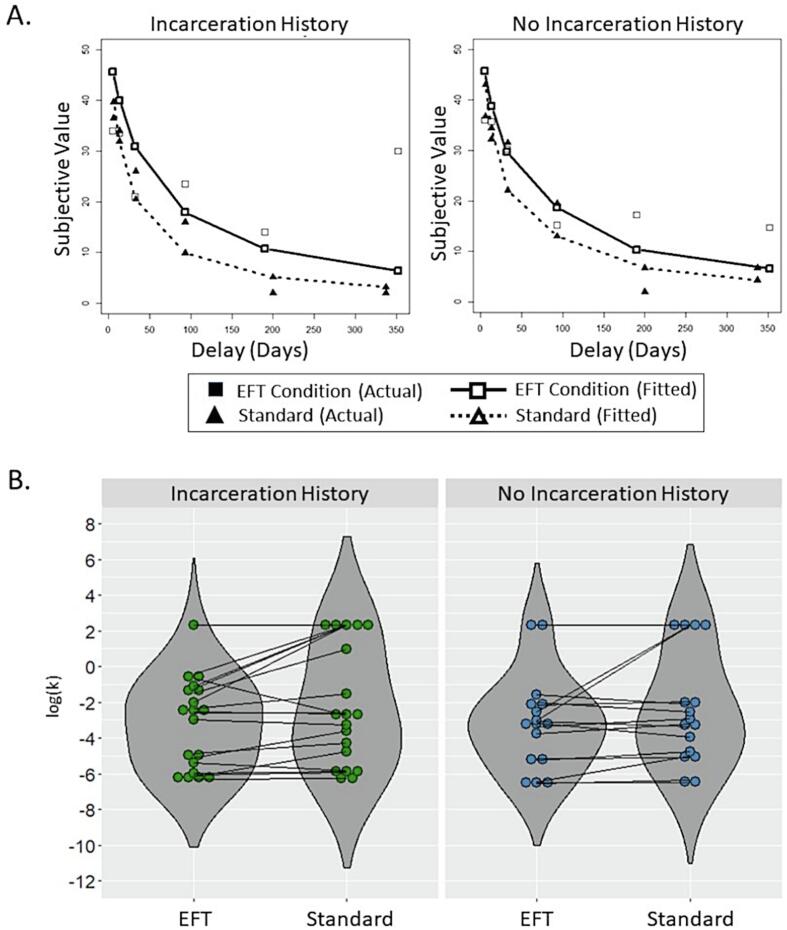
Plots depicting the median value of subjective reward by delay latency, as well as corresponding discounting functions and predicted values are presented separately for subgroups with and without a history of incarceration (A). A steeper discounting function is evident for Standard versus Episodic Future Thinking (EFT) conditions in both subgroups. Median values depicted were computed for the full sample and do not reflect omission of non-systematic responders. Individual values for the log-transformed discounting parameter (log(*k*)) by condition also reflect reduced discounting (i.e., lower values of log(*k*)) in EFT relative to Standard conditions for both subgroups (B). Violin plots summarize results by condition for individuals with (left) and without (right) a history of incarceration. Values for EFT and Standard conditions for each participant are presented as linked datapoints, without omission of non-systematic responders.

Discounting model fit was evaluated using root-mean-square-error and was comparable to that reported in our earlier work, and similar for EFT (*M* = 9.6, *SD* = 4.3) and Standard (*M* = 9.6, *SD* = 5.5) conditions, with no significant main or interaction effects identified through ART rANOVA. The same statistical approach was utilized for comparison of log(*k*) values and yielded a significant effect of condition (*F*(1,33) = 5.93, *p* = 0.02). The effect of incarceration history and interaction between condition and incarceration were non-significant. The significant effect of condition was driven by reduced discounting in EFT versus Standard conditions, as indicated by lower log(*k*) values, for those with and without a history of incarceration (see Fig. 1). However, within and between group *post hoc* comparisons returned no significant results, following correction for multiple comparisons.

Following exclusion of 4 non-systematic responders (see Supplementary Materials), the analysis again yielded a significant effect of condition only (*F*(1,29) = 6.06, *p* = 0.02). As before, log(*k*) values were lower for EFT relative to Standard for both subgroups but *post hoc* comparisons isolating the EFT effect within each subgroup did not survive correction for multiple comparisons. However, *post hoc* comparison of Standard discounting rates between groups yielded a significant result (*p* = 0.047, Tukey-adjusted), consistent with steeper discounting in the Standard condition for individuals with a history of incarceration.

## 4 Discussion

Considering EFT as an intervention to reduce impulsive decision-making, we hypothesized that EFT cues would significantly reduce DD in treatment-seeking individuals with CUD, regardless of incarceration history. Results support this hypothesis. While significantly steeper Standard discounting was identified in individuals with a history of incarceration when excluding non-systematic responders, DD did not differ when descriptors of personally-relevant future events were included. While requiring replication, our findings offer preliminary evidence that EFT manipulations can reduce discounting in individuals with CUD, regardless of personal incarceration history. Importantly, this was the case despite our relatively “hands-off” approach to evoking EFT through short textual cues describing events identified by participants. Previous work in clinical populations employed episodic specificity induction to prime or enhance elaborative mental simulation of future events and larger, more consistent EFT effects may be obtained through these methods (see, Forster, Steinhauer, et al., 2021, for additional discussion).

This work replicates our previous finding of the EFT effect in a pilot sample of 14 treatment-seeking individuals with CUD (Forster, Steinhauer, et al., 2021) and provides the first demonstration of reduced DD through an EFT manipulation wherein incarceration history has been specifically considered. Reactive, impulsive thought processes are a hallmark of criminal thinking that appears to be an instrumental link in the concatenation of predisposing and precipitating factors that lead to recidivism. While existing cognitive-behavioral interventions may serve to target reactive thinking (Walters, 2016), integrating EFT-based approaches could expand treatment options, especially in subpopulations with both criminal and substance use histories. EFT cues may be especially promising as just-in-time adaptive interventions to reduce impulsive decision-making in high-risk contexts for substance use and/or criminal behavior. Indeed, previous work in university students showed that EFT reduced hypothetical decisions to engage in delinquent behaviors (Wu et al., 2017) and such benefits may extend to real-world decisions with potential to save lives.

Beyond contributing novel evidence that the EFT effect is robust to incarceration history, the current work also adds to the literature by utilizing a Standard condition designed to more rigorously control for EFT framing effects. In our previous study (as in the seminal work of, Peters & Büchel, 2010), Standard delays included hashtags in place of textual cues used to describe future events in the EFT condition. This design, however, framed Standard delays as latencies, while EFT delays were framed as future reference points—failing to sufficiently control for a possible ‘date-delay framing’-like effect, wherein reduced discounting occurs when latencies are merely replaced with corresponding dates (DeHart & Odum, 2015). Our current findings suggest the EFT effect is present, even when controlling for ‘date-delay framing.’ Nevertheless, the current work is limited by its modest sample size and subtle differences in the EFT effect by incarceration history could emerge in larger samples with greater power to detect such effects. While this study cannot definitively rule out differences in the strength of the EFT effect by incarceration history, it serves to inspire future inquiry in this important area of research, which may also include consideration of current justice-involvement versus lifetime history.

## 5 Conclusions

Our findings replicate previous work demonstrating reduced DD in the context of EFT cues in individuals with CUD and further suggest this effect manifests in individuals with and without a history of incarceration. EFT-based interventions may therefore hold promise as a strategy to reduce impulsive decision-making in CUD, regardless of incarceration history.

## Role of funding sources

This work was supported by funding from IK2 CX001807/CX/CSRD VA (PI: Forster). The contents do not represent the views of the Department of Veterans Affairs, Department of Defense, or the United States Government.

## CRediT authorship contribution statement

**Taylor M. Torres:** Conceptualization, Investigation, Data curation, Writing – original draft. **Stuart R. Steinhauer:** Methodology, Writing – review & editing, Supervision. **Steven D. Forman:** Methodology, Writing – review & editing, Supervision. **Sarah E. Forster:** Funding acquisition, Conceptualization, Methodology, Software, Investigation, Data curation, Formal analysis, Validation, Visualization, Writing – original draft, Writing – review & editing.

## Declaration of Competing Interest

The authors declare that they have no known competing financial interests or personal relationships that could have appeared to influence the work reported in this paper.

## Data Availability

Data will be made available on request.
