# Supplementary material for: Patients with cocaine use disorder exhibit reductions in delay discounting with episodic future thinking cues regardless of incarceration history
Source: Addict Behav Rep. 2023 Oct 30;18:100518. doi: 10.1016/j.abrep.2023.100518 (PMC10632774; doi:10.1016/j.abrep.2023.100518)
Supplement: Supplementary data 1 [file mmc1.docx]

SUPPLEMENTARY MATERIAL FOR

***Patients with Cocaine Use Disorder Exhibit Reductions in Delay Discounting with Episodic Future Thinking Cues Regardless of Incarceration History***


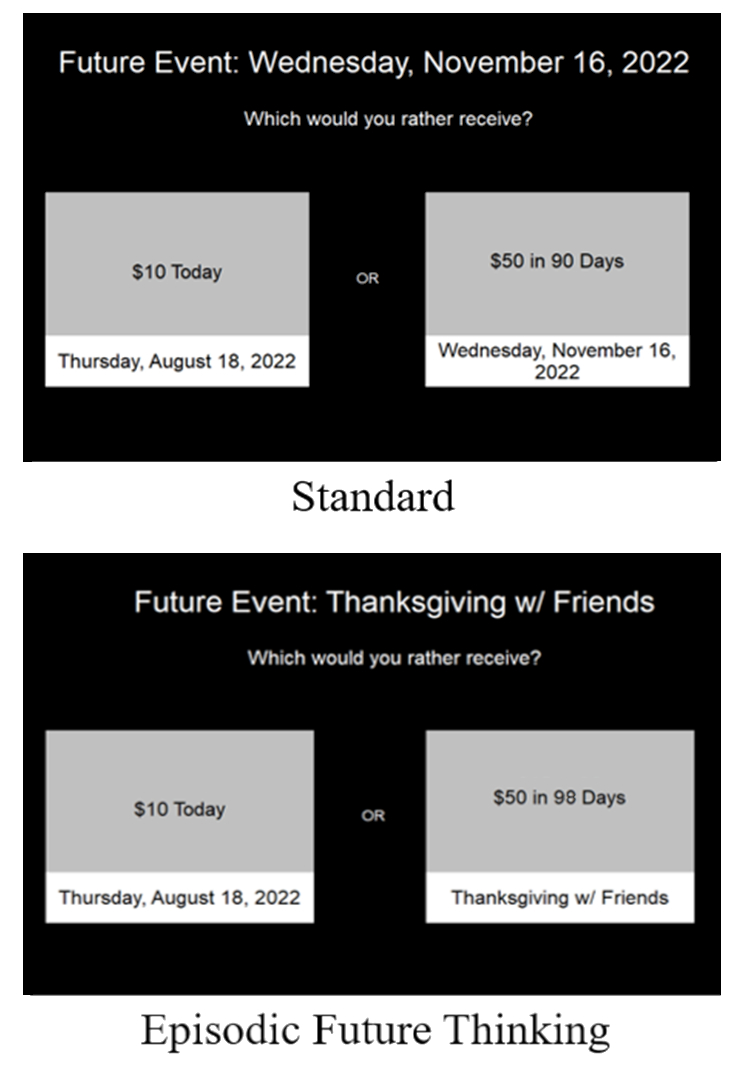
***Supplementary Figure 1.*** Example stimuli representing Standard (top) and Episodic Future Thinking (EFT) conditions in the personalized delay discounting task. In both conditions, participants were instructed to choose their preferred option by pressing the left- or right-most button on a Chronos (Psychology Software Tools, Pittsburgh, PA) response pad. The left button was used to a signal a preference for immediate reward and the right button was used to signal a preference for the delayed reward. In the EFT condition, an event cue was displayed at the top of the screen and below the larger, delayed reward. In contrast to our previous work (see Discussion), the date of the delayed reward was presented in place of event cues on Standard trials to control for differences in ‘date-delay framing’ between conditions that could contribute to the EFT effect.

***Rating Scale Anchors***

Events referenced in the personalized delay discounting task were rated on a 7-point Likert scale for personal relevance, valence, and arousal/excitement. Anchors provided for Likert scale ratings were as follows: personal relevance (1 = “not at all relevant”, 7 = “extremely relevant”), valence (1 = “totally neutral”, 7 = “extremely positive”), and arousal/excitement (1 = “not at all excited”, 7 = “extremely excited”).

***Detection of Non-Systematic Response Patterns***

A modified version of the algorithm developed by Johnson & Bickel (2008) to support detection of non–systematic response patterns in delay discounting was implemented. However, it is noted that cues included to evoke Episodic Future Thinking (EFT) may introduce non–systematic response patterns by altering the response context across target latencies. While we attempted to identify personally–relevant future events for use in the EFT condition that resulted in comparable ratings for relevance, positive valence, and excitement/arousal across target latencies for each participant, it is still possible that particular event cues had a stronger impact on decision–making, introducing additional variability independent of delay. Similarly, the inclusion of event cues in the EFT condition could also potentially impact decision–making in the Standard condition by introducing temporal context information that alters processing of Standard latencies. Consequently, response patterns in the current paradigm may be more variable than contexts involving standard delay discounting only and results were therefore reported with and without exclusion of these data.

Because EFT manipulations may result in data that violate the assumption of monotonically decreasing subjective value with increasing delays employed in the Johnson & Bickel (2008) algorithm, this method was specifically applied to data from the Standard condition. Participants exhibiting non–systematic response patterns in the Standard condition were then removed from the analysis such that both EFT and Standard data were omitted. Two modifications to the Johnson & Bickel (2008) algorithm were additionally applied to accommodate the relatively low maximum reward value ($50) and short–term maximum delay latencies (approximating one year) used in the current paradigm. Specifically, Criterion 1of the algorithm—which evaluates for unexpected increases in subjective value across consecutive delay windows—utilized a threshold of 30% of the maximum reward value (i.e., $15) rather than the 20% threshold used for a maximum value of $1,000 (i.e., $200). Criterion 2 was additionally applied to ensure that the subjective value of the maximum delay latency was less than the subjective value of the minimum delay latency by at least 10% the maximum reward value (i.e., $5). However, this criterion was only applied when there was sufficient variability in subjective value over the six target latencies (*SD* > 10) to avoid exclusion of participants with consistently high or low valuation of delayed reward across latencies.
